# Scaffolder - software for manual genome scaffolding

**DOI:** 10.1186/1751-0473-7-4

**Published:** 2012-05-28

**Authors:** Michael D Barton, Hazel A Barton

**Affiliations:** 1 Biology Department, The University of Akron, Akron, OH, 44325-3908, USA

## Abstract

**Background:**

The assembly of next-generation short-read sequencing data can result in a fragmented non-contiguous set of genomic sequences. Therefore a common step in a genome project is to join neighbouring sequence regions together and fill gaps. This scaffolding step is non-trivial and requires manually editing large blocks of nucleotide sequence. Joining these sequences together also hides the source of each region in the final genome sequence. Taken together these considerations may make reproducing or editing an existing genome scaffold difficult.

**Methods:**

The software outlined here, “Scaffolder,” is implemented in the Ruby programming language and can be installed via the RubyGems software management system. Genome scaffolds are defined using YAML - a data format which is both human and machine-readable. Command line binaries and extensive documentation are available.

**Results:**

This software allows a genome build to be defined in terms of the constituent sequences using a relatively simple syntax. This syntax further allows unknown regions to be specified and additional sequence to be used to fill known gaps in the scaffold. Defining the genome construction in a file makes the scaffolding process reproducible and easier to edit compared with large FASTA nucleotide sequences.

**Conclusions:**

Scaffolder is easy-to-use genome scaffolding software which promotes reproducibility and continuous development in a genome project. Scaffolder can be found at http://next.gs.

## Background

High-throughput sequencing can produce hundreds of thousands to millions of sequence reads from a genome. At the time of writing, high-throughput sequencing is limited to producing reads less than 1,000 nucleotides in length. Therefore to resolve a sequence longer than this, such as a complete genome, these numerous smaller fragments must be pieced together. The process of joining reads into longer sequences is the ‘assembly’ stage of a genome project [1].

Assembly software takes the nucleotide reads produced by sequencing hardware and, in the ideal case, outputs a single complete genome sequence composed of these individual fragments. An analogy for this process is a jigsaw puzzle: each nucleotide read represents a single piece, and the final genome sequence is the completed puzzle. Sequences of repetitive nucleotide ‘repeat’ regions or biased and incomplete sequencing data may prevent the genome being assembled into a continuous sequence. This may be due to insufficient or multiple different overlaps between reads and is analogous to missing pieces in the jigsaw or pieces that fit to multiple other pieces.

The advent of high-throughput sequencing methods has led to a renewed interest in algorithms to solve the problem of genome assembly [2,3]. The complexity of merging large numbers of overlapping reads can lead to genome assembly software being unable to produce a complete sequence. Instead, the algorithm may generate several large assembled regions of sequence (‘contigs’) composed of the many individual reads. These contigs represent a fragmented picture of the genome and therefore require additional work to join together into a complete sequence.

The process of finishing a genome sequence can be expensive in terms of time and laboratory effort. In some cases the genomic data present in a set of generated contigs may be sufficient for many research questions [4]. Nevertheless, a continuous high-quality ‘finished’ genome sequence does provide a greater depth of information, such as complete resolution of repeat regions and precise estimates of distances between genomic elements [5,6]. The process of joining these contigs together to form a continuous genome sequence is called the ‘scaffolding’ or ‘finishing’ stage and is the focus of the software described in this article.

## Scaffolding

Scaffolding is the process of joining a series of disconnected contigs into a complete continuous genome sequence. Due to genomic complexity and missing data, scaffolding may not ultimately produce a final completed sequence, but may still succeed in joining a subset of contigs together or resolving gaps between contigs. An overview of the required steps in the scaffolding process is outlined below:

## Contig orientation

The sequencing process generates reads from either strand of the DNA helix and the resulting contigs constructed from these reads may represent either DNA strand. Orientating all contigs to point in the same direction requires reverse complementing sequences where necessary. In the case of archaeal and bacterial genomes this orientation will be to the 5’ → 3’ direction following the direction of genome replication.

## Contig ordering

Contig ordering determines the placement of observed contigs to best represent their order in the true genome sequence. The correct placement of each contig also highlights any extra-genomic DNA, such as plasmids which are scaffolded separately from the genomic sequence. The order is commonly started at the contig containing the origin of replication. All subsequent contigs are then ordered in the 5’ → 3’ direction of DNA replication.

## Contig distancing

Given the correct order and orientation, determining the distance between contigs results in an estimate of the complete genome size. The size of any inter-contig gaps represents the length of an unknown region in the genome. Filling these regions with unknown nucleotide characters ‘N’ allows a draft continuous sequence. This sequence is useful for representing both the known and to-be-resolved areas in the genome sequence.

## Gap closing

During the scaffolding process, closing and filling gaps between contigs completes and improves the genome scaffold. Closing gaps may require returning to the laboratory to perform additional sequencing or using computational methods to estimate the unknown sequence. This additional sequence is used to replace the gap between two contigs, joining them into a single sequence. Once all contigs have been joined and gaps in a scaffold closed, the genome may be considered finished.

## Computational methods for scaffolding

The process of finishing a genome scaffold uses wet laboratory methods, in silico methods, or a combination of both. An example of a computational method might use the paired-read data from the sequencing stage. The occurrence of paired reads in separate contigs can be used to estimate probabilistically the order and distance between these contigs. Alternatively, laboratory methods may use PCR to amplify the unknown DNA in a gap region then use Sanger sequencing to determine the sequence of this gap. Computational methods, using available sequencing data, are preferable as they are less costly in laboratory time and materials compared to manual gap resolution [7]. Finally when the scaffold cannot be completely resolved, in silico software packages exist to suggest the likely primers necessary for PCR amplifying the sequence in gap regions [8].

Examples of in silico methods include comparing the assembled contigs to a complete reference genome sequence to search for areas of sequence similarity between the two. Any areas of corresponding sequence in the reference genome can be used to infer contig placement and build the contigs into a scaffold [9-11]. Genomic recombination can however reduce the efficacy of this. Repeat regions may also be responsible for multiple gaps when building a genome sequence; tandemly repeated nucleotide regions in the genome produce multiple reads with similar sequence. As many assembly algorithms rely on sequence overlaps between reads to build a contig, the similarity between repeat-region reads can result in the assembly collapsing into an artificially short sequence or being ignored by more conservative assembly algorithms. Such regions can be resolved by using algorithms that specifically reassemble the collapsed repeat region [12,13]. A related approach uses unassembled sequence reads matching the regions around a scaffold gap to construct a uniquely overlapping set of reads across the gap. [14].

Paired-read data can provide an extra level of information about how contigs may be scaffolded together. Heuristic scaffolding algorithms take advantage of this data to search for the optimal configuration of contigs in the scaffold that matches these paired-read distances [15,16]. Synteny data from a reference genome can also be combined with this paired-read data to estimate the best contig configuration [17].

These described in silico methods provide a wide array of approaches for merging contigs into a larger, continuous scaffold sequence. The scaffolding process may still require manually inserting additional sequences or further joining contigs using PCR-derived sequence. Moving and editing large blocks of nucleotide text by hand however introduces human error and precludes any reproducibility.

The software outlined here, “Scaffolder,” aims to address these problems of reproducibility by creating a file syntax and software framework for editing a genome scaffold. Scaffolder uses a specific file format to define how contigs are joined, additional sequences are inserted, and for the specification of unknown regions. This syntax allows a scaffold to be updated by simply editing the scaffold file. As such, Scaffolder facilitates a reproducible finishing process and provides a concise overview of how the final genomic scaffold was constructed.

## Implementation

### Code and dependencies

Scaffolder is written in the Ruby programming language and tested against versions 1.8.7 and 1.9.2 [18]. The Scaffolder package is split into two libraries. The first called “scaffolder” which provides the core Scaffolder application programming interface (API). The second library “scaffolder-tools” provides the Scaffolder command line interface (CLI).

Unit tests were implemented to maintain individual elements of the source code during development and were written using the Shoulda and RSpec [19] libraries. Integration tests were written to test the Scaffolder software interface as a whole and were written using the Cucumber library [19].

The Scaffolder source code is documented using the Yard library [20]. Unix manual pages for the command line were generated using the Ronn library [21]. The manipulation of biological sequences in Scaffolder uses the BioRuby library [22]. A full list of the software dependencies in Scaffolder can be found in the Gemfile in the root of each source code directory.

### Scaffold file syntax

The choice of nucleotide sequences comprising the scaffold is specified using the YAML syntax [23]. YAML is a data format using whitespace and indentation to produce a machine readable structure. As YAML is a standardised data format, third-party developers have the option to generate a genome scaffold using any programming language for which a YAML library exists. The YAML website lists current parsers for languages including C/C++, Ruby, Python, Java, Perl, C#/.NET, PHP, and JavaScript. In addition to being widely supported, YAML-formatted scaffold files can be validated for correct syntax using third-party tools such as Kwalify [24].

Initial sequencing data assembly may result in an incomplete genome build. Adding further sequences from either PCR or computational methods also means that genome scaffolding may be an on-going process. The scaffold file should therefore be simple to update manually in addition to being computationally tractable. This requirement was also best suited to YAML syntax which is human-readable and simple to edit in a standard text editor.

The scaffold file takes the form of a list of entries. Each entry corresponds to a region of sequence used in the final scaffold sequence. Each entry in the scaffold file may have attributes that define whether a sub-sequence or the reverse complement of the sequence should be used. The types of attributes available, and an example scaffold file are outlined in the Results section.

The input data for Scaffolder are nucleotide sequences in FASTA format file. These nucleotide sequences can be of any length and may be individual reads, assembled contigs or contigs which have been joined into larger scaffolds. The case in which Scaffolder may be most useful is using the contigs and scaffolded contigs, combined with additional gap filling sequences produced by PCR or in silico methods as outlined in the Background.

## Results

### Scaffolder simplifies genome finishing

The Scaffolder software facilitates reproducibly joining nucleotide sequences together into a single contiguous scaffolded super-sequence. Plain-text scaffold files written in YAML specify how these sequences should be joined. The Scaffolder software is used to generate the scaffold sequence from these instructions. In addition to specifying which contigs are required, the scaffold file allows the contigs to be edited into smaller sub-sequences or reverse complemented if necessary. Each scaffold file represents one scaffolded nucleotide sequence and as such separate scaffolds should be defined in separate files.

The process of genome finishing may involve producing additional oligonucleotide sequences to fill unknown regions in a scaffold. The Scaffolder format provides functionality to use these additional insert sequences to fill gaps. These inserts can also be treated in the same manner as larger contig sequences: trimmed and/or reverse complemented to match the corresponding gap region size and orientation.

The distances between contigs may be estimated from paired-read data or from mapping the contigs to a reference genome. These inter-contig gap regions are useful to join separate sequences together by the estimated distance. The scaffold file allows for the specification of such unresolved regions by inserting regions of ‘N’ nucleotides into the scaffold. The use of these regions in the scaffold indicates the unresolved regions in the build and their approximate size.

The nucleotide sequences used in the scaffold are maintained as a separate FASTA file: the nucleotide sequences are referenced in the scaffold using the first word from the FASTA header of the corresponding sequence. Maintaining the nucleotide sequences in a separate file preserves the unedited sequence and decouples the data from the specification of how it should be used to produce the genome sequence.

### Defining a scaffold as a text file

**Figure 1:**
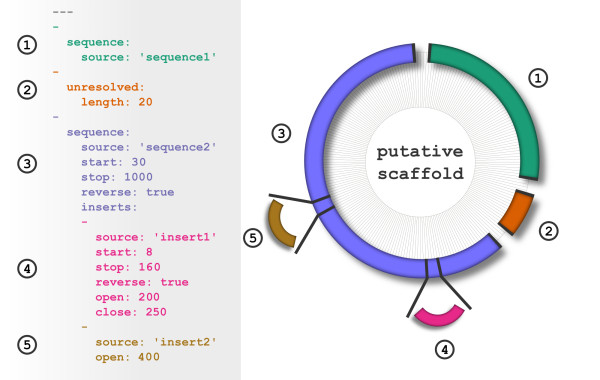
**Example of Scaffolder File and the Resulting Build An example scaffold file written using the YAML syntax**[1]**(left) and the resulting putative scaffold sequence (right).** The scaffold contains three entries and two inserts. Each entry in the scaffold file text is delimited by a ‘-’ on a new line and highlighted using separate colours. The scaffold diagram on the right is not to scale and instead illustrates how the scaffold sequences are joined.

The scaffold file is written using the YAML syntax and an example is shown in Figure 1. This file illustrates the text attributes used to describe a scaffold and how the sequences are correspondingly joined together in the genome build. The basic layout of the scaffold file is a list of entries, where each entry corresponds to a region of sequence in the generated scaffold super-sequence.

### Simple sequence region

The first line of the scaffold file in Figure 1 begins with three dashes to indicate the start of a YAML-formatted document. The first entry (highlighted in green) begins with a dash character ‘-’ to denote an entry in the YAML list. This is a requirement of the YAML format: each entry begins with a dash line. The next line is indented by two spaces where whitespace is used to group similar attributes together. The “sequence” tag indicates that this entry corresponds to a sequence in the FASTA file and the following line indicates the name of this sequence using the “source” tag. The first word of the FASTA header is used to identify which sequence is selected from the file. Together these three lines describe the first entry in the scaffold as a simple sequence using a FASTA entry named ‘sequence1’. On the right hand side of Figure 1 this produces the first region in the scaffold, also shown in green.

### Unresolved sequence region

The second entry in the scaffold, highlighted in orange, is identified by the “unresolved” tag, indicating a region of unknown sequence but known length. The second line specifies the size of this unknown region. In this example this entry produces a region of 20 ‘N’ characters in the scaffold.

### Trimmed sequence region with multiple inserts

The last entry in the scaffold, highlighted in blue, adds a FASTA entry named ‘sequence2’ to the scaffold. This entry demonstrates how this sequence may be manipulated prior to addition to the scaffold. The ‘start’ and ‘stop’ tags trim the sequence to these coordinates inclusively. The “reverse” tag also instructs Scaffolder to reverse complement the sequence. In the putative scaffold shown in Figure 1 this completes the sequence.

This final entry in the scaffold uses the “inserts” tag to add additional regions of sequence. These inserts are also added as a YAML list, with each insert beginning with a dash. The first insert, shown in purple, uses similar attributes to that of a sequence entry; the reverse, start and stop tags are used to trim and reverse complement the insert. Similarly the ‘source’ tag identifies the corresponding FASTA sequence as ‘insert1’. The “open” and “close” tags are specific to inserts and determine where the insert is added in the enclosing sequence. The region of the sequence inside these coordinates is inclusively replaced by the specified insert sequence. This is visualised in the putative scaffold in Figure 1 by the black lines bisecting the blue sequence.

The next insert, shown in brown, is specified using only the ‘open’ tag. This illustrates that only one of either the ‘open’ or ‘close’ tags is required when adding an insert sequence. If only one of the ‘open’ or ‘close’ tags is used the corresponding opposing ‘open’/‘close’ coordinate is calculated from the length of the insert FASTA sequence. This allows inserts to bridge into, and partially fill, gap regions without requiring an end coordinate position.

### Scaffolder software interface

Scaffolder provides a standardised set of Ruby classes and methods (API) for interacting with the scaffold. This allows Scaffolder to be integrated into existing genomics workflows or used with Ruby build tools such as Rake. In addition Scaffolder provides a command line interface (CLI) to validate the scaffold file and build the draft super sequence. The Scaffolder CLI behaves as a standard Unix tool and returns appropriate exit codes and manual pages. The use of both these Scaffolder interfaces is outlined in detail on the Scaffolder website (http://next.gs). This website provides a “getting started” guide as an introduction to using Scaffolder to build a genome scaffold.

## Discussion

Scaffolding an incomplete genome assembly requires joining contigs and additional gap-filling sequences using a combination of computational and laboratory methods. The process of manually editing a scaffold is inherently hard to reproduce and introduces irreproducible edits and/or human error. In respect to this the aims of the Scaffolder software are twofold: 1) to provide software that is easy to install and simplifies the task of genome finishing; and 2) to facilitate reproducibility in the scaffolding and finishing stage of a genome project. Scaffolder uses a minimal and compact syntax to describe how a genome scaffold sequence should be generated. This syntax is simple to write and edit whilst being succinct and readable.

AGP is a similar format for describing scaffolds. This format can be used to describe contig order and N-filled gap regions in a scaffold. The advantage of the AGP format is that each contig entry is defined on a single line which allows searching the scaffold using Unix line-based tools. The Scaffolder format in contrast is written in the standardised YAML format and therefore accessible to the many languages which provide parsers to this format. The Scaffolder format is provided with a tool explicitly to produce the FASTA sequence of scaffold specified by the file. The Scaffolder format further provides functions for trimming and replacing regions of sequence using inserts.

Constructing a genome by specifying the scaffold organisation in text file makes generating a scaffold super sequence both reproducible and deterministic for the same file and set of FASTA sequences. In comparison, joining large nucleotide sequences by hand cannot be reliably reproduced, while the scaffold file also provides a human readable description of how the scaffold is constructed. Configuring the final sequence in the scaffold file means the build is easier to edit, once constructed.

An example use case for Scaffolder is a combination of computational and manual editing of a genome scaffold. We have used Scaffolder in our own genome projects to create an initial scaffold from computationally parsing the output of in silico scaffolding tools into YAML. This scaffold was then manually updated as the scaffold was finished with additional gap-filling sequences generated in the laboratory. This is example of the Scaffolder format being both computationally tractable while being simple to edit manually. The YAML text format also allows comparison of differences between scaffold builds using standard Unix tools such as diff. This therefore makes scaffold files amenable to storage in version control systems and allows genome finishers to use methods similar to those in software development.

## Conclusions

Scaffolder is software, written in Ruby, aimed at both bioinformaticians and biologists familiar with the command line who wish to build a genome scaffold from a set of nucleotide sequences. The Scaffolder file format maintains the genome scaffold as a concise and readable text representation that allows third-parties to see how the genome sequence was scaffolded. This file format also allows a broad overview of which sequences were included and how they are ordered in the genome scaffold, something not possible to deduce from a megabase-length string of nucleotide characters. Scaffolder furthers increases the ease of reproducibility in genome projects by allowing the scaffold super-sequence to be reliably reproduced from the same scaffold file. The YAML syntax for writing the scaffold file is also standardised and simple to manipulate programmatically. This thereby means the scaffolding process follows the Unix tenet of “If your data structures are good enough, the algorithm to manipulate them should be trivial.”

## Availability and requirements

**Project name:** Scaffolder v0.4.4, Scaffolder Tools v0.1.3

**Project home page:**http://next.gs

**Operating system:** Platform Independent. Tested on Mac OS X and Ubuntu. Programming language: Ruby 1.8.7 or 1.9.2

**Other requirements:** RubyGems package management software and the following libraries: BioRuby 1.4.x, confligliere 0.1.x, ronn 0.7.x. A full list of development dependencies can be found in the Gemfile in the base directory of each project.

**License:** MIT

**Any restrictions to use by non-academics:** None

## Abbreviations

API, Application programming interface; CLI, Command line interface; PCR, Polymerase chain reaction; YAML, YAML ain’t markup language [23].

## Competing interests

The authors declare no competing interests.

## Authors’ contributions

MDB developed and maintains the Scaffolder tool. MDB and HAB wrote the manuscript. All authors have read and approved the manuscript.
